# Supplementary material for: Genome-Wide Identification and Expression Analysis of the Starch Synthase Gene Family in Sweet Potato and Two of Its Closely Related Species
Source: Genes (Basel). 2024 Mar 25;15(4):400. doi: 10.3390/genes15040400 (PMC11049646; doi:10.3390/genes15040400)
Supplement: Supplementary file 1 [file genes-15-00400-s001.zip › genes-2891145-supplementary.pdf]

Table S1 The primer sequences for qPCR of sweet potato *SS* genes.

Primer name	Forward Primer（5’→3’）	Reverse Primer（5’→3’）
IbSS1	**TGTGGACATTTTGAAGCGCA**	**AACTCTCCATAGCAACCGCC**
IbSS2	**GCCTGTGAAGCTCCGTTAGT**	**CATTGTGCGGGCGATACTTG**
IbSS3	**CCTTGCAAAAGCACCTTGGG**	**ACAAACTGGAGACCCCTCCT**
IbSS4	**AACGTGTGAAAGCTACGGGT**	**TCTTTCAGCAATCTGTCGGAGT**
IbSS5	**TCAAGGAAACTGGAGAACGCA**	**TCTTCCTGAGATGCAGCAAGTT**
IbSS6	**CGAGGCACCGAGAGTTTTGA**	**GAAAGCAACCTTGGCGTTCA**
IbSS7	**GCAATAAAGCGGGTGGAGGA**	**AGGATACGGATGCTGGAACC**
IbSS8	**GGAGGCCTTGGTGATGTTGT**	**AGTTCCACCCCAAGAGTAGC**
IbARF	**CTTTGCCAAGAAGGAGATGC**	**TCTTGTCCTGACCACCAACA**
